# Secondary Syphilis Rash

**DOI:** 10.31662/jmaj.2023-0068

**Published:** 2023-09-13

**Authors:** Shun Takei, Kiyozumi Suzuki, Hiromasa Otsuka, Seishi Watanabe

**Affiliations:** 1 Department of General Medicine, Ageo Central General Hospital, Saitama, Japan

**Keywords:** secondary syphilis, *Treponema pallidum*, skin rash, palms and soles

A 57-year-old heterosexual male with diabetes was admitted to our hospital for glycemic control. During his physical examination, a nonpruritic rash was found on his trunk and extremities, including palms and soles (Figure 1). He reported a previous genital lesion and a similar rash that resolved spontaneously. Six months earlier, he had unprotected sex with a casual female partner. He was diagnosed with secondary syphilis based on being positive for rapid plasma reagin and *Treponema pallidum* hemagglutination. The test for human immunodeficiency virus was negative. He was treated with amoxicillin, which resulted in the healing of the rash.

**Figure 1.**
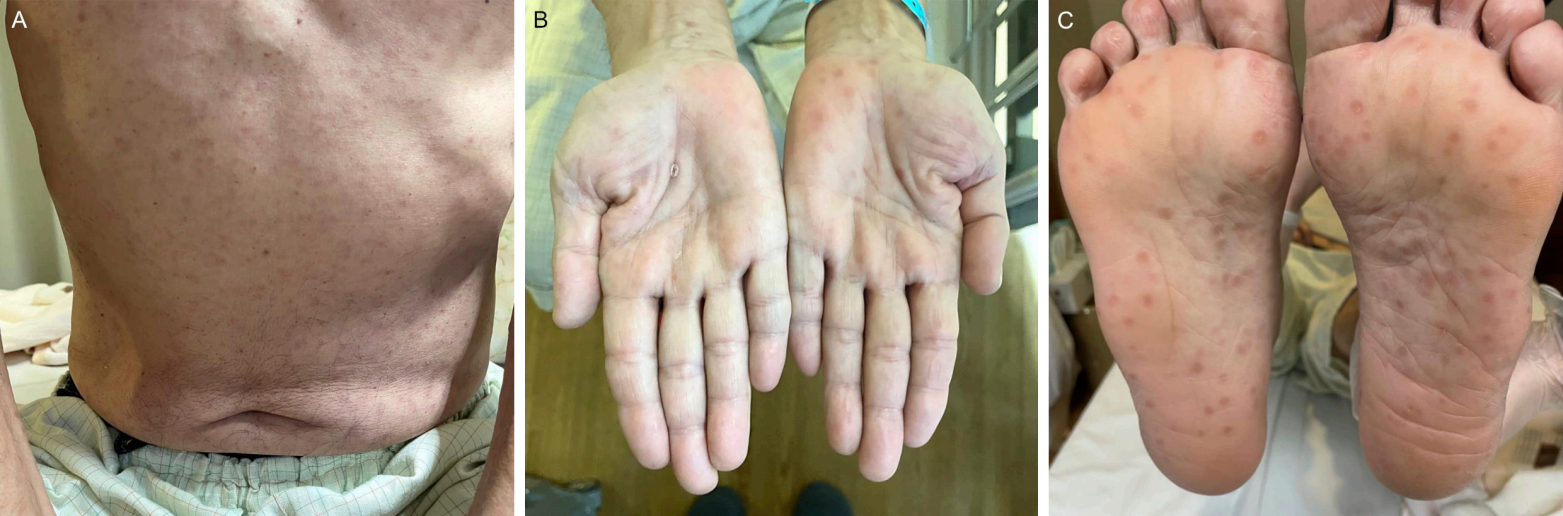
Diffused erythematous maculopapular rash on the trunk (A). Multiple rounded erythematous or reddish-brown spots on the palms and soles, partly with scale (B, C).

The rash of secondary syphilis is typically diffuse, nonpruritic, symmetric, and maculopapular on the trunk and extremities; however, the rash is nonspecific and difficult to distinguish from other skin rashes ^(1), (2)^. Nonetheless, the rash involving the palms and soles is a crucial clue to the diagnosis of secondary syphilis ^(2)^. Considering the rapid increase in the number of syphilis cases in Japan ^(3), (4)^, physicians should conduct a thorough dermatological assessment, including the examination of the palms and soles, to avoid overlooking the diagnosis of syphilis.

## Article Information

### Conflicts of Interest

None

### Author Contributions

Shun Takei: Writing - Original draft, Methodology

Kiyozumi Suzuki: Methodology, Writing - review and editing

Hiromasa Otsuka: Methodology, Writing - review and editing

Seishi Watanabe: Methodology, Writing - review and editing

All authors critically reviewed the manuscript.

### Informed Consent

Consent was obtained from the patient for the use of images for publication.

### Approval by Institutional Review Board (IRB)

In this study, IRB approval was not required.
